# No correlation between triage of the patient in the Emergency Department and time before first doctors note at the main medical ward

**DOI:** 10.1186/1757-7241-20-S2-P33

**Published:** 2012-04-16

**Authors:** Mads Dannesbo, Søren Wistisen Rasmussen, Thomas Andersen Schmidt

**Affiliations:** 1 The Emergency department, Holbaek Hospital, Denmark

## Background

In the Emergency Department (ED) admitted patients are triaged in accordance with how urgent medical treatment they need.

What happens to the patients after initial treatment at admission? Is there a correlation between degree of acute illness in patients and when the follow up on the ward will occur?

We focused on patients admitted with medical problems which is the main part (~60%) of patients admitted to hospital. Most of patients with medical problems are referred to “Akut Visitationsafsnit” (AVA), and therefore the study is based on AVA patients. In this study we used the patient triage on arrival as an indicator of acute illness.

## Methods

We used OPUS (a hospital management information system) to determine the time that passed from completion of the medical chart upon arrival in the ED to the first written follow up note in the chart from an AVA physician, in relation to the patients’ triage in the ED from 1 (the most ill) to 4. Critically ill patients were admitted to an Intensive Care Unit. The study design was retrospective with data from October 2010, which gave us 227 observations.

To take multiple comparisons into account one-way analysis of variance was performed to assess overall significance, which was followed by a Tukey standardized range test procedure to locate the possible differences. This two-step procedure controlled the experiment-wise error rate at a 5% significance level.

## Results

We found no correlation between triage of the patient and time before first note on AVA and no significant difference between the 4 subgroups.

## Conclusion

Presumably the most ill patients will require the most intensive treatment, but the triage may be a poor indicator of patients with need of medical intervention after initial treatment and stabilizing. Intervention by doctors may be less needed and initial plan and treatment may be sufficient until follow-up the next day. Patients in subgroup 3 and 4 do seldom require immediate medical attention, but are mainly admitted for diagnostic reasons.

